# Optimal design for longitudinal studies to estimate pubertal height growth in individuals

**DOI:** 10.1080/03014460.2018.1453948

**Published:** 2018-04-18

**Authors:** Tim James Cole

**Affiliations:** Population, Policy and Practice Programme, UCL Great Ormond Street Institute of Child Health, London, UK

**Keywords:** Height, puberty, age at peak velocity, SITAR

## Abstract

**Background:** The SITAR model expresses individual pubertal height growth in terms of mean size, peak height velocity (PHV) and age at PHV.

**Aim:** To use SITAR to identify the optimal time interval between measurements to summarise individual pubertal height growth.

**Subjects and methods:** Heights in 3172 boys aged 9–19 years from Christ’s Hospital School measured on 128 679 occasions (a median of 42 heights per boy) were analysed using the SITAR (SuperImposition by Translation And Rotation) mixed effects growth curve model, which estimates a mean curve and three subject-specific random effects. Separate models were fitted to sub-sets of the data with measurement intervals of 2, 3, 4, 6, 12 and 24 months, and the different models were compared.

**Results:** The models for intervals 2–12 months gave effectively identical results for the residual standard deviation (0.8 cm), mean spline curve (6 degrees of freedom) and random effects (correlations >0.9), showing there is no benefit in measuring height more often than annually. The model for 2-year intervals fitted slightly less well, but needed just four-to-five measurements per individual.

**Conclusions:** Height during puberty needs to be measured only annually and, with slightly lower precision, just four biennial measurements can be sufficient.

## Introduction

Growth in puberty is recognised as an important stage in the life course, with the pattern of growth at this time impacting significantly on later health. As a recent example, pubertal height and weight growth in the British 1946 Birth Cohort were found to have a clinically important effect on bone health 50 years later (Cole et al. 2016; Kuh et al. 2016). It was not only the individual’s size (i.e. mean height and weight) in puberty that was influential, but also two aspects of their pubertal growth spurt: its timing and intensity.

The timing of puberty based on height or weight in individuals is usually defined by the age when they are growing fastest, their age at peak velocity (Marshall & Tanner 1969, 1970). Equally, the intensity of puberty is summarised by their peak velocity, which is inversely correlated with their duration of puberty—the faster they grow, the sooner they reach adult size. The mean age at peak height velocity (PHV) is ∼13–14 years in boys and 2 years earlier in girls, while mean PHV is 8–9 cm/year in the two sexes. However, both timing and intensity vary enormously from one individual to another; the population standard deviations (SD) of PHV and age at PHV being ∼0.9 cm/year and 1 year, respectively (Cole et al. 2014). Thus, the distribution of age at PHV in the population spans more than 4 years in each sex.

In the past, the age at peak velocity in individuals was estimated by fitting a growth curve to their serial growth data, e.g. the five-parameter Preece-Baines curve (Preece & Baines 1978) or a natural cubic smoothing spline curve (Sandhu et al. 2006). From this their age at peak velocity could be obtained, either as the solution to a quadratic in the parameters or from the first derivative of the spline curve. However, the downside of this approach was the need to repeat the analysis for each individual.

Now, with the advent of non-linear mixed effects models, it is possible to estimate puberty timing for individuals by fitting a single model to the entire cohort, and functions of the subject random effects identify the timings for individuals. A recent paper by Simpkin et al. (2017) compared three such methods for estimating age at PHV: a multilevel model using fractional polynomials (MLM-FP) (Goldstein 1986), superimposition by translation and rotation (SITAR) (Cole et al. 2010) and principal analysis by conditional expectation (PACE) (Yao et al. 2005). Each model was compared with the Preece-Baines model 1 fitted to individuals (Preece & Baines, 1978) and the focus was the degree of bias in the estimated mean age at PHV. The authors concluded that, of the three models, only SITAR estimated age at PHV, essentially without bias, and they recommended it and the Preece-Baines model on this basis.

The SITAR model (Cole et al. 2010) has a number of advantages over the Preece-Baines model. It fits all the individuals in a single model, whereas Preece-Baines fits them separately. It estimates the age at PHV in individuals as a random effect relative to the mean age at PHV, which is simpler to obtain than calculating it from other parameters. It also fits the mean curve as a cubic spline, which is more flexible in shape than the parametric Preece-Baines curve. In addition, SITAR includes two further subject-specific random effects representing each individual’s relative size and growth intensity (i.e. PHV), which, together with the timing random effect, account for most of the variance in pubertal height growth—SITAR consistently explains over 98% of the cross-sectional variance (Cole et al. 2010, 2014, 2015; Prentice et al. 2012).

The analysis by Simpkin et al. (2017) simulated height growth curves for boys from 10–19.75 years using the Preece-Baines model 2 (Preece & Baines 1978), while systematically varying the measurement error, sample size, time interval between measurements, and measurement balance. It found that SITAR performed well, irrespective of the selected measurement error, sample size, or time interval, with the exception of measurements every 24 months when the model failed to converge or was seriously biased. Simpkin et al. (2017) also analysed the same four models using real data for boys from Christ’s Hospital School, and found that the correlations between age at PHV estimated by the different methods were generally low.

In their analyses Simpkin et al. (2017) varied the frequency of measurement occasions, but they did not address the specific design question as to which measurement interval is the most efficient for estimating pubertal growth, and in previous cohort studies this interval has varied widely. J. M. Tanner’s Harpenden Growth Study collected measurements every 3 months during puberty (Marshall & Tanner 1969, 1970), while the Edinburgh Growth Study saw subjects every 3 or 6 months (Cole et al. 2014), the Wrocław Growth Study collected measurements annually (Bielicki & Waliszko 1975), and the Avon Longitudinal Study of Parents And Children (ALSPAC) clinically examined children every 1 or 2 years during puberty (Boyd et al. 2013). It is evident that more frequent measurements will lead to better estimates, be they of the timing or the intensity of the growth spurt. However, there is likely to be an optimal measurement frequency, such that extra measurements beyond the optimum improve the estimates only marginally.

This study aims to use the SITAR model to address the question: what is the optimal time interval between measurements to quantify pubertal height growth in individual boys, providing adequate accuracy at reasonable cost? In doing so it will build on and extend the work of Simpkin et al. (2017), using the same Christ’s Hospital School dataset.

## Methods

### Data management

The analysis is based on a single dataset of 129 508 height measurements collected on 3245 boys aged 9–19 years from Christ’s Hospital Boys School, Horsham, Sussex, UK, between 1939 and 1968 (heights were not measured in the CH Girls School). Christ’s Hospital is an independent school, established in 1552, which has always admitted pupils from a wide social and geographical background, asking parents to contribute towards fees according to their means. Virtually all the boys were born in the UK, so it is likely the vast majority were of European descent. Simpkin et al. (2017) used broadly the same dataset, with 126 897 heights from 3123 boys.

Height was measured with an Avery yard-arm platform scale with height standard attached and recorded to the nearest 1/8^th^ inch (3.2 mm) (Friend 1935). For analysis, the heights were expressed in centimetres to two decimal places. The boys were measured at the start and end of each term, i.e. six times a year, throughout their time at the school. The first height per child was at a median age of 10.6 (interquartile range = 9.8–11.3) years, the last height was at 17.5 (16.6–18.3) years, and the time interval from first to last measurement was 6.8 (5.8–7.8) years. The scale of the dataset makes it suitable to compare the performance of different sampling designs.

The dataset was first cleaned by excluding measurements whose residuals after fitting the SITAR model (see next section) exceeded 4 residual standard deviations (3.1 cm) in absolute value (*n* = 382 or 0.3%). The same dataset, except with 952 outlying measurements excluded, was used to validate the SITAR growth curve model, see Figure 3(c) of Cole et al. (2010).

The ideal dataset to address the research question would consist of frequent regularly spaced measurements, from which longer measurement intervals could be obtained by selecting suitably spaced sub-sets of the data. However, the school pattern at Christ’s Hospital consisted of a shorter spring term, its length depending on the timing of Easter, and longer summer and autumn terms, while the holidays were of 4 or 8 weeks duration. As a result, the distribution of time intervals between measurements had three distinct peaks at 5, 9 and 12 weeks. This complicated the selection of suitable measurements to represent particular time intervals. In addition, some measurements were inevitably missing, so it was not realistic to expect every *n*^th^ measurement to be equally spaced in time. Instead, an algorithm was developed to extract equally spaced measurements, which has been implemented as the *timegap* function in the *sitar* library (Cole 2016) of the statistical language *R* version 3.3.2 (R Core Team 2016). The same library was used to fit the SITAR models.

Timegap works as follows: (a) a target time interval is specified (e.g. 6 months); then, for each individual, timegap (b) calculates the differences in age between all pairs of measurements; (c) expresses them as multiples of the target interval; (d) restricts them to those within a tolerance of 10% of (an integer multiple of) the target interval; and (e) identifies those providing the longest sequence of measurements. In this way sub-sets of measurements for each individual were obtained where the time gaps between measurements were close to one or more target intervals.

The following time intervals were investigated as simple fractions or multiples of 12 months: 2, 3, 4 and 6 months, 1 and 2 years, and sub-set datasets for each interval were constructed using timegap. The 2-year interval was included to test the findings of Simpkin et al. (2017). Note that the actual ages of measurement were not constrained. Time intervals of 3, 4 and 5 years were also explored, but the SITAR models fitted to them failed to converge and they are not considered further. Inevitably the numbers of subjects and measurements in each dataset differed, and, to address this, the data were restricted to the sub-set of individuals with measurements in all six datasets.

### Statistical analysis

The purpose of the analysis was to compare the different time intervals by summarising the data using a separate SITAR model for each interval; the model based on the full dataset was also fitted for comparison. SITAR (SuperImposition by Translation And Rotation) is a shape invariant growth curve model consisting of a natural cubic B-spline mean curve and three subject-specific random effects that have the effect of shifting the mean curve to match the subjects’ own growth curves (Beath 2007; Cole et al. 2010; Lindstrom 1995). The three random effects are *size*, a measure of relative height in the individual, *timing* (or *tempo),* the relative timing of their age at PHV (APHV), and *intensity*, their relative PHV. In geometric terms the three random effects can be thought of as *size* shifting the individual curves up/down, *timing* shifting them left/right, and *intensity* stretching/shrinking the age scale, to alter the average slope. The purpose of these adjustments is to—as closely as possible—superimpose the individual curves on the mean curve. The complexity of the mean spline curve’s shape was controlled by choosing the degrees of freedom to minimise the Bayesian Information Criterion (BIC).

The SITAR models were fitted, as height vs log age, with either 5 or 6 degrees of freedom for the mean curve, age being log-transformed to reflect a multiplicative age effect. With log age the timing random effect is a difference in log age, which corresponds to a percentage difference in age (Cole & Altman 2017). So individuals differ in developmental age by a few percentage points, which corresponds to the age scale being stretched or shrunk; this is more valid biologically than a linear age scale being shifted right or left.

The model included both random and fixed effects for size, timing, and intensity. The size effect was in units of centimetres, while timing and intensity were in fractional units (i.e. multiplied by 100 to give percentages) relative to APHV and PHV, respectively. The models were compared on the basis of mean curve shape, residual standard deviation, mean APHV (the age when the first derivative of the mean curve, plotted as height vs age, was maximal), mean PHV (the velocity at mean APHV) and the random effect standard deviations and their correlations across the models for different time intervals.

To investigate the 2-year interval in more detail, separate models were fitted for individuals with two, three, four and five measurements.

## Results

**Figure 1.**
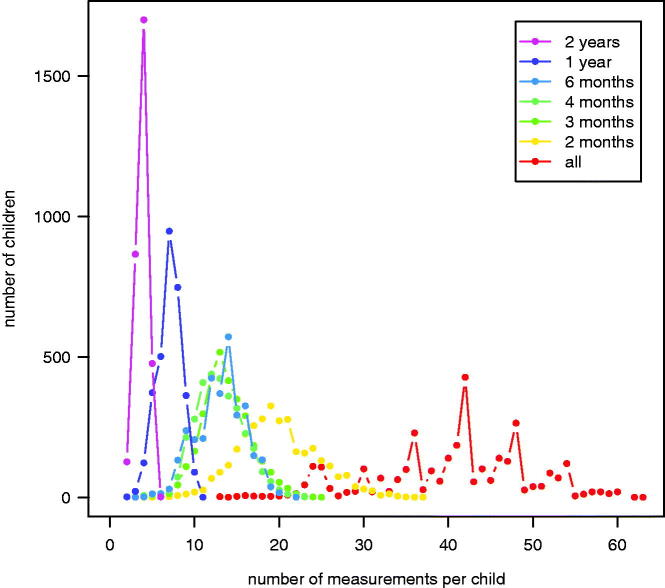
Plot of the numbers of measurements per child vs the number of children in each time interval dataset. For example, 1700 children each had four measurements in the 2-year interval dataset (purple), while 428 had 42 measurements in the full dataset (red).

The edited dataset consisted of 3172 individuals and 128 679 measurements (99.4% of the total), age range = 9.0–20.5 years (median = 14.0, interquartile range = 12.2–15.8). Figure 1 shows the numbers of children with specified numbers of measurements for the different time intervals, e.g. for 2-year intervals each of the 3172 boys had between two and six measurements, with a mode of four.

SITAR models were successfully fitted to the whole dataset and to the sub-sets of data for the six time intervals from 2 months to 2 years. All the models converged with 6 degrees of freedom, except for the 2-year model, which converged with 5.

Table 1 compares summary statistics for the data and fitted SITAR models based on all the data and the six time intervals. The numbers of measurements per dataset correspond to the areas under the corresponding curves in Figure 1. In theory, the numbers should be inversely related to the time intervals, with proportionately more measurements for shorter intervals as seen for 6 months, 1 and 2 years. However, the intervals for 2, 3 and 4 months were under-represented due to the non-uniform termly measurement pattern. The modal numbers of measurements per child and the proportions of measurements spaced the nominal time interval apart (the remainder being two or more intervals apart) confirm that many of the 2- and 3-month measurements were actually spaced 4 or 6 months apart.

**Table 1.** Summary statistics for datasets and fitted SITAR models for the six time intervals in 3172 boys.

Time interval	Number of heights	Number of heights per child (mode)	Proportion of time gaps of one time interval (%)	Degrees of freedom for spline curve	Residual standard deviation (cm)	Variance explained (%)	Mean age at peak height velocity (years)	Mean peak height velocity (cm/year)	Size random effect SD (cm)	Timing random effect SD (%)	Intensity random effect SD (%)
All	128 679	42	—	6	0.74	98.7	14.4	9.6	6.0	6.6	15
2 months	62 965	19	41	6	0.76	98.7	14.4	9.5	6.0	6.6	14
3 months	43 542	13	22	6	0.77	98.7	14.4	9.5	5.9	6.5	14
4 months	41 074	12	54	6	0.77	98.7	14.4	9.6	6.0	6.6	14
6 months	41 504	14	96	6	0.77	98.6	14.4	9.6	5.9	6.6	14
1 year	22 237	7	99.5	6	0.80	98.5	14.4	9.7	5.8	6.5	14
2 years	12 049	4	99.9	5	0.92	98.0	14.2	9.7	5.7	6.2	14

The last eight columns of Table 1 summarise the SITAR models. The residual standard deviation rose very slightly with increasing time interval and the percentage of variance explained fell slightly, while the mean ages at PHV and mean PHVs were very similar across models, as were the standard deviations (SD) of the size, timing and intensity random effects. Multiplying the timing SDs by median age converts them to age units of 0.9 years, slightly less than the expected 1 year.

**Figure 2.**
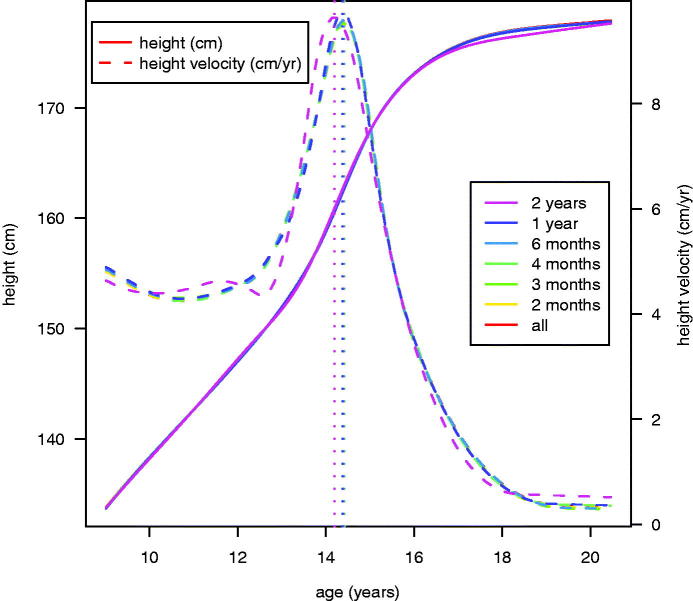
Plot of mean height curves (solid lines) and mean height velocity curves (dashed lines) based on SITAR models for the different time intervals. The vertical dotted lines indicate the age at peak velocity for each model.

Figure 2 shows the fitted mean height and height velocity curves overall and for the six intervals. The seven curves were remarkably similar, with those for all, 2, 3 and 4 months almost completely obscured by the 6-month and 1-year curves on top of them. As already seen in Table 1, their mean ages at peak velocity (marked with the vertical dotted lines) were also virtually identical, averaging 14.4 years with a range of only 0.03 years (11 days).

The 2-year model differed somewhat from the others, with a larger RSD, less variance explained, an earlier mean age at PHV, larger mean PHV, and smaller random effect SDs (Table 1). However, the differences were not large. Its height and height velocity curves also differed in shape (Figure 2).

**Figure 3.**
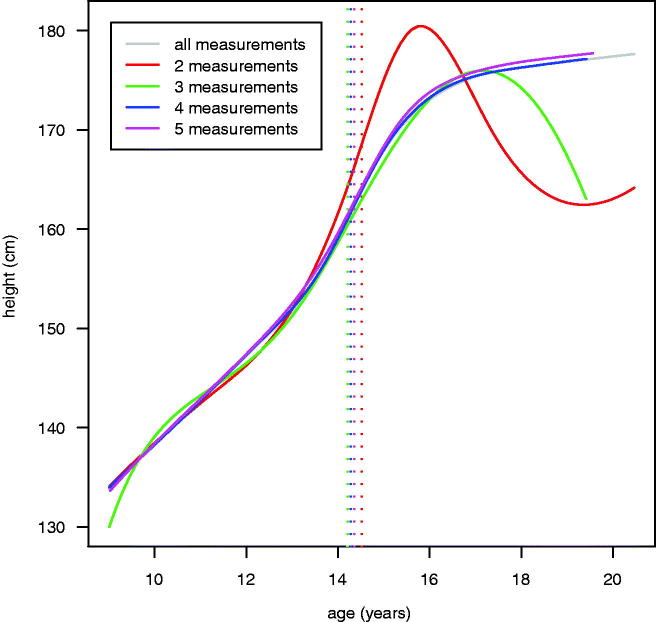
Plot of mean height curves for SITAR models fitted to sub-sets of the 2-year interval dataset, based on the number of measurements per individual. The vertical dotted lines indicate the age at peak velocity for each model.

However, the fact that the 2-year model fitted at all was somewhat surprising, with a median of only four measurements per child (note that Simpkin et al. (2017) also had trouble fitting the 24-month model). Table 2 compares the 2-year time interval models restricted to individuals with two, three, four and five measurements. At first glance they agree well and even those based on two and three measurements per child look plausible. However, in detail the 2- and 3-measurement models were unreliable; the former only fitted with the timing random effect omitted, and the latter did not converge, both due at least in part to their young ages of measurement (see Table 2). Figure 3 shows the separate mean curves for the four models, which confirms that at least four measurements were needed to provide reliable mean curves, corresponding to 6 years of follow-up. This latter group contained 1700 (54%) of the 3172 individuals and a further 477 (15%) had five measurements, so their curves were robustly estimated. Note that the overall 2-year curve is effectively the weighted average of the curves in Figure 3.

**Table 2.** Summary statistics for 2-year time intervals: separate SITAR models according to the number of heights per child.

Number of heights per child	Number of children	Median age (years)	Proportion of time gaps of one time interval (%)	Degrees of freedom for spline curve	Residual standard deviation (cm)	Variance explained (%)	Mean age at peak height velocity (years)	Mean peak height velocity (cm/year)	Size random effect SD (cm)	Timing random effect SD (%)	Intensity random effect SD (%)
2	127	11.7	99.2	4	0.93	98.1	14.5	14.3	5.0	—*	2
3	866	13.4	99.7	4	0.95	98.0	14.2	8.3	5.8	10.9	13
4	1700	14.1	100	5	0.89	97.7	14.3	9.4	5.6	6.2	14
5	477	14.0	100	5	0.97	97.6	14.4	9.1	5.7	6.4	13
6	2	—	—	—	—	—	—	—	—	—	—

* Timing random effect omitted.

**Figure 4.**
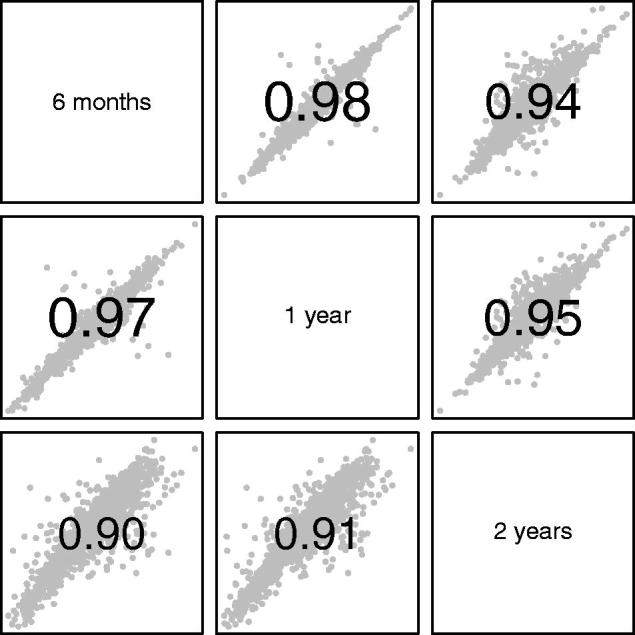
Scatterplot matrix of random effects for size (upper triangle) and timing (lower triangle) in SITAR models restricted to 6-month, 1-year and 2-year time intervals. The corresponding correlation coefficients are also shown. To read off the values for a given pair of time intervals, focus on the rows and columns containing the two names; there are two cells where they intersect, the one above the diagonal containing the size correlation and the one below the corresponding timing correlation.

**Figure 5.**
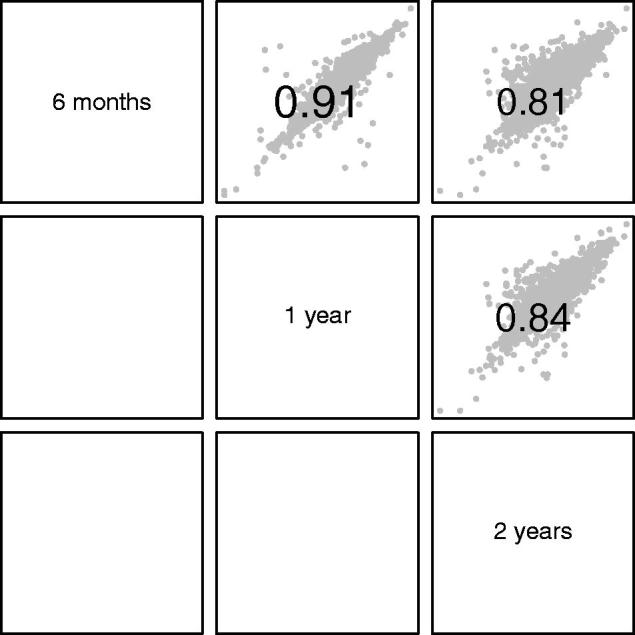
Scatterplot matrix of random effects for intensity in SITAR models restricted to 6-month, 1-year and 2-year time intervals. The corresponding correlation coefficients are also shown.

Figures 4 and 5 compare the subject random effects as estimated by the different models, by looking at scatterplot matrices of the correlations between them. Due to the similarity of the models from 2 months to 6 months, the comparison is restricted to intervals of 6, 12 and 24 months. Figure 4 covers size and timing, while Figure 5 deals with intensity. Each individual graph shows the scatterplot of a pair of individual random effects (in grey), with the value of the correlation coefficient superimposed.

The correlations for size and timing between the models with different time intervals (Figure 4) all exceeded 0.9, showing good agreement between the models, although slightly smaller for 2 years. For intensity (Figure 5) the correlations were smaller but still exceeded 0.8. Thus, the models for the different time intervals all provided reassuringly similar estimates for the subject random effects, particularly age at peak velocity. This in turn confirmed the value of the 1- and 2-year interval models.

## Conclusion

On the evidence presented here, measurements in puberty taken 1 year apart are as informative as measurements taken 2 months apart for estimating the timing and intensity of peak height velocity in individuals. This insight could reduce the annual number of measurements in future studies from six to one, with little loss of precision—clearly a considerable saving. Extending the time interval to 2 years would further halve the number of measurement occasions, but at a cost of slightly greater imprecision. Ideally the median age of measurement (14 years here) should be close to mean APHV.

The fact that the 2-year interval model fitted as well as it did is instructive. The modal and median number of heights per boy was four (range is two-to-six), and the total number of heights only 9% of that for the full model, yet the two estimated mean ages at PHV differed by only 0.2 years and the two SDs for the timing random effect, at 6.2% and 6.6%, were also very similar. It seems to be a good example of ‘borrowing strength’, that just a few measurements per child spread evenly across the age range can accurately estimate the timing of puberty (Cole et al. 2016).

With Simpkin et al. (2017) the 24-month model failed, fitting poorly for the unbalanced design (each measurement age within a 3-month window) and failing to fit at all for the balanced design (all individuals measured at the same ages). Why this should be is unclear, as Simpkin et al. (2017) gave no details of the models they fitted, e.g. the number of spline degrees of freedom. However, Table 1 provides a clue, at least for the unbalanced case, in that here the 2-year model fitted with 5 degrees of freedom, but not 6. Simpkin’s model might well have fitted had they used fewer degrees of freedom.

The study by Simpkin et al. (2017) involved both simulated and real data, the simulations testing for bias in APHV while the Christ’s data explored model agreement in APHV. The findings here rely on real data, which were obviously less tightly controlled than simulated data. However, they were largely equally spaced in time, thanks to timegap, and the distribution of measurement ages reflected those likely to be seen in practice. Thus, the findings have face validity.

It has long been known that estimating the shape of the mean curve in puberty without adjusting for the pattern of growth in individuals attenuates the velocity curve and biases the mean curve (Cole et al. 2008; Merrell 1931). By adjusting for individual timing and intensity, SITAR provides an unbiased estimate of the curve, irrespective of the number of measurements. In this sense, the findings are unsurprising, that reducing the number of measurements (and increasing the time interval between them) hardly affects the mean curve (and hence APHV), as confirmed by Simpkin et al. (2017). However, the fact that the residual standard deviation and the random effects are also largely unaffected is an unexpected bonus.

How generalisable are the findings? It is impossible to know without repeating the study in different contexts, but several previous studies have shown that pubertal height growth in both sexes is well modelled by SITAR (Cole et al. 2010, 2014, 2015; Prentice et al. 2012). As such, it efficiently estimates the variability in the age at PHV, so it is well suited to compare different designs for estimating it. A limitation of the current dataset is its being restricted to boys, and the pubertal growth spurt is known to be less intense in girls (Marshall & Tanner 1969), which might make its timing harder to estimate. Against that the standard deviation of APHV is similar in the two sexes (Marshall & Tanner 1969, 1970; Cole et al. 2014), which suggests that the findings should generalise. Note that, because girls start puberty 2 years earlier than boys on average (Marshall & Tanner, 1969), their measurement ages need to be advanced correspondingly.

The strengths of the study are the application of SITAR growth curve analysis to efficiently summarise the information available for each measurement interval and the large number of subjects and measurements over an age range exceeding 6 years, allowing comparison of time intervals from 2 months to 2 years. Limitations include the data being restricted to boys, as discussed above, the ethnicity, and the data being rather old, dating back 50–80 years. These factors are likely to affect mean age at PHV to some extent, but not the paper’s main conclusions. In addition, the non-uniform measurement pattern through the year has meant that the shorter time intervals were under-represented compared to those for 6 months or more.

The topic of optimal design for longitudinal studies as addressed here has an extensive literature, which has tended to focus on designed missingness (Helms, 1992; Morara et al. 2007; Verbeke & Lesaffre, 1999). This corresponds to the mixed longitudinal design, where individuals are recruited and followed up from different ages, as opposed to the pure longitudinal design of the present study. However, the question addressed here could be extended to include designed missingness by excluding individuals from further measurement once they have clearly passed their age at peak velocity. This would reduce the number of measurements needing to be collected later in puberty, although whether or not the saving was worthwhile would need further work.

In conclusion, the SITAR analysis has demonstrated that annual measurements are sufficient to estimate age at peak height velocity to high precision, and that biennial measurements on as few as four occasions suffer only a slight loss of precision.
